# Hypoglycaemic Principles of *Securidaca longepedunculata* Extracts Reverses Experimental Diabetes and Diabetes Associated Pathology in Wistar Albino Rats

**DOI:** 10.1155/bri/7173019

**Published:** 2025-07-31

**Authors:** Obiora Emmanuel Abonyi, John Onyebuchi Ogbodo, Simeon Ikechukwu Egba, Innocent Ogheneovo Orhonigbe, Obioma Uzoma Njoku, Okwesili Fred Chiletugo Nwodo

**Affiliations:** ^1^Department of Medical Biochemistry College of Medicine, Enugu State University of Science and Technology, Agbani, Enugu, Nigeria; ^2^Department of Science Laboratory Technology, University of Nigeria, Nsukka, Nigeria; ^3^Department of Biochemistry, Kampala International University, Kampala, Uganda; ^4^Delta State University, Abraka, Delta State, Nigeria; ^5^Department of Biochemistry, University of Nigeria, Nsukka, Nigeria

**Keywords:** alloxan, hypoglycaemic, oral glucose tolerance test, *Securidaca longepedunculata*, Wistar rats

## Abstract

**Background:** This study investigated the effect of the extractable hypoglycaemic principles from *Securidaca longepedunculata* leaves on some biochemical parameters in alloxan-induced diabetic rats with the view to having improved pharmacological agent(s) that could be used in managing diabetes mellitus with relatively lesser side effects. Three groups of four rats each were used for the oral glucose tolerance test (OGTT): group one received 3 mL/kg BW normal saline (normal control) while groups 2 and 3 received 200 and 400 mg/kg BW of methanol extract of *Securidaca longepedunculata* (MESL), respectively. Six groups of five rats per group were used for the main antidiabetic study. Group one received 3 mL/kg BW normal saline (normal control), group 2 received no treatment after induction, group 3, received 5 mg/kg BW glibenclamide (standard group), groups 3–6 received 100, 200, and 400 mg/kg BW MESL, respectively, after induction.

**Results:** The OGTT showed significant (*p* < 0.05) reduction in random blood sugar of the treated groups compared to the control. In the antidiabetic study, the treated groups 4–6 significantly (*p* < 0.05) lowered the blood glucose of the Wistar rats compared to the treatment control. Extract administration showed no adversity to liver and kidney, respectively, as shown by result of tested standard indicators. Equally MESL caused significant (*p* < 0.05) increase in serum antioxidant activities and significant (*p* < 0.05) decrease in malondialdehyde concentration compared to the treatment control.

**Conclusion:** The study suggested hypoglycaemic potency, antioxidant rejuvenation, and relative hepatic and nephron safety by MESL.

**Summary**

• The extract (MESL) significantly reduced random blood sugar in diabetic rats.
• Administration of MESL helped experimental rats to overcome oxidative stress caused by diabetes.
• Administration of MESL did not produce toxicity to both liver and kidney in rats confirming their safety.

## 1. Introduction

Diabetes mellitus is a wasting disorder of carbohydrate metabolism characterized by sustained elevated circulating glucose molecules in the blood stream. The preponderance of glucose in the blood and attendant paucity in the cells lead to a wide range of life-threatening complications eventually leading to death if not well managed. Starved of glucose molecules in a near permanent state, a lot of metabolic activities are imperiled [1]. Diabetes mellitus has been reported as one of the major causes of different complications and deaths worldwide [2–4]. Two types of diabetes mellitus abound, namely juvenile and late-onset, respectively [5]. While insulin therapy is ideal for type I, oral hypoglycaemic principles are most suitable for type II. The oral hypoglycaemic principles currently available are sulfonylureas, biguanides, α-glucosidase inhibitors, etc. [6, 7]. These hypoglycaemic principles are known to have different shortcomings ranging from beta-cell depletion, hypoglycemia, and monotherapy-inadequacy to general-synthetic-drugs shortfalls [8, 9].

Certain biochemical parameters are used to determine the integrity of hepatocytes. Elevated value of these markers in the blood is an established indicator of organ injury or damage as seen in disease conditions. Alanine aminotransferase (ALT) and aspartate aminotransferase (AST) are veritable marker enzymes for heart and liver injury or diseases [10, 11]. Alkaline phosphatase (ALP) is also a hydrolytic enzyme whose unusual increase in the blood signals tissue damage. ALP together with ALT and other similar enzymes form dependable diagnostic tool for liver injury and disease [12, 13].

Diabetes is the leading cause of established renal failure in the Western world [14] one of the most important determinants of kidney function is creatinine level. Its value is estimated by the rate of production of creatinine which is in a function of muscle mass [15]. It is advised that people with diabetes should regulate their blood pressure thereby slowing down the progression of chronic kidney disease. This is important since normal levels of serum electrolytes are needed to maintain normal pressure [16]. It has been shown that diet modification can lead to decrease in postprandial glucose levels and reductions in medications among diabetics. It has proved very useful in the management of pregnancy-related hyperglycaemia and adequate buffer against diabetes-related complications for unborn babies of diabetic mothers.

Different plants have demonstrated abilities to reduce high blood glucose which is the family of Polygalaceae and has been credited for ameliorating various illnesses [17]. However, there is scanty evidence, on the hypoglycaemic characteristics of the plant. Hence, this work was designed to evaluate hypoglycaemic principles extractable from the plant on some biochemical parameters in diabetic Wistar rats with the view to getting a biological principle with concomitant lesser side effects.

## 2. Methodology

### 2.1. Preparation of *Securidaca longepedunculata* Powder

The leaves were obtained from a natural habitat in Opi Nsukka. It was identified and assigned identification number (CEDD/1600) in Bioresources Diversity and Conservative Programme (BDCP) Nsukka by Mr. Alfred Ozioko.

#### 2.1.1. Extraction of *S. longepedunculata* Constituents

Plant leaves were dried in air pulverized. A quantity (1200 g) of the pulverized leaves were macerated in 3 L of 80% methanol for 72 h and then filtered. Concentration the filtrate was done using a rotary evaporator (Edward England) at 45°C and the solid extract was stored in the refrigerator until used.

### 2.2. Animal Experiments

Male Wistar albino rats (120–150 g) were procured from certified breeders at Faculty of Veterinary Medicine, University of Nigeria, Nsukka (UNN). They were acclimatized for 7 days maintained on regular feed (rat chow) and clean water *ad libitum*. The animals were housed in aluminum cages in clean conditions at ambient room temperature of 27°C with 12 h light and 12 h dark cycle. The Principles of Laboratory Animal Care (NIH, 1985) guidelines were followed throughout the duration of this study. The study was approved by the Chairman of Ethics Committee, on the recommendation of Faculty of Biological Sciences Research Ethics Committee (FBSREC), UNN with certificate reference number: UNN/FBS/23/PhD/2017/246868B.

### 2.3. Determination of the Effect of Methanol Extract of *S. longepedunculata* (MESL) on Oral Glucose Tolerance Test (OGTT)

The OGTT was done on MESL according to the method of Eyth et al., [18]. Twelve rats weighing 120–150 g were grouped into three groups. The animals fasted for 18 hours. Their fasting blood sugar (FBS) was determined using an Accu-Chek glucometer. Group one was given 3 mL/kg BW normal saline while groups two and three received 200 and 400 mg/kg BW of MESL, respectively. Thirty minutes later, 40% glucose weight per volume (w/v) was administered to all the groups at a dose of 1 mL/100 g body weight orally. Their blood glucose levels were monitored at 30, 60, and 120th minutes after the glucose administration.

### 2.4. Determination of Hypoglycaemic Potency of MESL

Thirty albino rats were divided into six groups of five rats. Group one was given normal saline (3 mL/kg BW) without induction, while groups two to six were all treated with 100 mg/kg BW of alloxan, but group two was not treated with either extract or standard drug. While groups three to six were given 5 mg/kg BW glibenclamide and 100, 200, and 400 mg/kg BW of MESL, respectively. Hyperglycaemia was confirmed 48 h after alloxan administration. Thereafter, treatment began and the random blood sugar (RBS) levels were checked on day 3, day 6, and day 9. A cardiac puncture was used to collect the blood from the rats for different biochemical analyses.

### 2.5. Determinations of ALT and AST Activities

ALT and AST were determined using the method of Reitman and Frankel [19] as outlined in Teco Kit.

### 2.6. Determination of Bilirubin Concentration

The concentrations of total and conjugated bilirubin were determined using the method of Jendrassik and Grof [20] as outlined in Randox Kit.

### 2.7. Determinations of Urea and Creatinine Concentrations

The concentrations of urea and creatinine were evaluated with the method of Bartels and Bohmer [21] as described in Randox Kit.

### 2.8. Determination of Electrolytes

The electrolytes, sodium, potassium, chloride, and bicarbonate ions, respectively, were measured using standard spectrophotometric methods [22–26].

### 2.9. Assay of Antioxidant Enzymes and Determination of Lipid Peroxidation Marker

Activities of antioxidant enzymes were assayed as described by Xin et al. [27]; Aebi, [28], and Paglia and Valentine [29], respectively, while malondialdehyde concentration was determined using the method described by Wallin et al. [30].

### 2.10. Statistical Analysis

Data obtained were presented as means ± SEM. One-way analysis of variance (ANOVA) was used to further analyze the generated data. Separation of means was done using Duncan's multiple test. Significantly different results were accepted @ *p* ≤ 0.05 with statistical package for service solutions (SPSS) version 20.

## 3. Results

### 3.1. Effects of Crude MESL on OGTT

The FBS of the groups showed relatively normal levels of glucose (71.75–95.67 mg/dL). After 60 min of the administration of 40% glucose solution, the RBS of the test groups and control reached their peaks (125.33–140.25). At the 120^th^ minute, varying degrees of reduction were seen. The difference between the 120^th^ minute and the FBS at zero (0) minute showed significant (*p* < 0.05) reduction in RBS of the treated groups two and three compared to the control group one as shown in Table 1.

### 3.2. Effect of MESL on Mean Glucose Levels of Treated Albino Wistar Rats

On day three the treated groups 3 (standard drug), 5 (200 mg/kg BW MESL), 6 (400 mg/kg BW MESL) gave nonsignificant (*p* > 0.05) decrease in RBS, while group 4 (100 (treatment control). At day 6, a significant (*p* < 0.05) reduction in RBS was observed in groups three, four, and six while group five showed a nonsignificant (*p* > 0.05) increase in RBS when compared to that of treatment group two. On day 9, there was a significant (*p* < 0.05) reduction in RBS in both (treated) groups 3 and 4 compared to the treatment control, whereas there was a nonsignificant (*p* > 0.05) reduction in both groups 5 and 6 compared to the treatment control as presented in Table 2.

### 3.3. Effect of MESL on Mean Serum ALT, AST, and ALP Activities of Treated Albino Wistar Rats

The result of the liver marker enzyme ALT showed a significant (*p* < 0.05) decrease in activity in the treated groups 3 and 6 when compared to group 1 while groups four and five indicated (*p* < 0.05) increase in ALT activity compared to that of group two. But there existed a significant (*p* < 0.05) increase in ALT activity of MESL treated groups 4 and 5 compared to the standard drug (glibenclamide).

There was a significant (*p* < 0.05) decrease in AST activity in treated groups 3–6 compared to group 2 and a nonsignificant (*p* > 0.05) to the standard drug.

**Figure 1:**
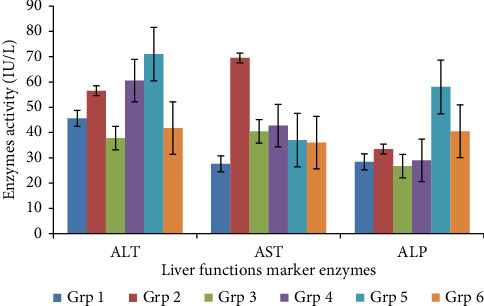
Effect of methanol extract of *Securidaca longepedunculata* leaves on the mean serum alanine aminotransferase, aspartate aminotransferase, and alkaline phosphatase activities (U/L) Group 1: no induction + 3 mL/kg BW (normal control). Group II: induced with alloxan, but no treatment. Group III: induced with alloxan + 5 mg/kg BW of glibenclamide. Group IV induced with alloxan + 100 mg/kg BW of MESL. Group V: induced with alloxan + 200 mg/kg BW of MESL. Group VI: induced with alloxan + 400 mg/kg BW of MESL.

The ALP gave nonsignificant (*p* > 0.05) reduction in groups 3 and 4 while groups five and six indicated a significant (*p* < 0.05) increase in ALP activities compared to the normal control as shown in Figure 1.

### 3.4. Effect of MESL on Mean Serum Total and Conjugated Bilirubin Concentrations of Treated Albino Wistar Rats

The standard group three showed a nonsignificant (*p* > 0.05) decrease in total bilirubin levels while groups four, five, and six indicated a nonsignificant (*p* > 0.05) increase in total bilirubin concentrations compared to group 1. However, there was a significant (*p* < 0.05) increase in total bilirubin concentrations in each of groups 3–6 compared to treatment group 2. Also, there were nonsignificant (*p* > 0.05) differences in total bilirubin concentrations of MESL treated groups compared to group 3.

There was significant (*p* < 0.05) increase in conjugated bilirubin levels in groups three to six compared to the normal and treatment controls. The MESL treated groups four and five indicated a significant (*p* < 0.05) increase while group six produced a significant (*p* < 0.05) decrease in conjugated bilirubin concentration compared to the standard drug as shown in Table 3.

### 3.5. Effect of MESL on Mean Serum Electrolytes Concentrations of Treated Albino Wistar Rats

The sodium ion concentration of treated groups 3, 5, and 6 produced a significant (*p* < 0.05) increase compared to the control. Group four indicated a significant (*p* < 0.05) decrease in sodium-ion level compared to the treatment control. The MESL treated groups 4-6 showed nonsignificant (*p* > 0.05) differences in Na^+^ concentration compared to the standard drug.

The treated groups three to six demonstrated a nonsignificant (*p* > 0.05) increase and nonsignificant (*p* > 0.05) decrease in the concentration of serum K^+^ compared to the normal and untreated controls, respectively.

Chloride ion levels of groups 3–6 demonstrated nonsignificant (*p* > 0.05) differences in concentrations compared to normal and untreated controls. Bicarbonate ion levels of groups 3–6 demonstrated nonsignificant (*p* > 0.05) differences in bicarbonate ion concentrations compared to normal and untreated controls, respectively, as presented in Table 4.

### 3.6. Effects of MESL on Mean Serum Urea and Creatinine Concentrations of Treated Albino Wistar Rats

The urea concentrations of groups 3–6 produced a significant (*p* < 0.05) rise compared to the control group. While groups 3–6 indicated a significant (*p* < 0.05) reduction in urea level compared to the untreated group.

Groups 3–6 showed a nonsignificant (*p* > 0.05) rise in creatinine concentration compared to the normal control. Groups 3–6 demonstrated significant (*p* < 0.05) reduction in creatinine concentration compared to the untreated control. There was nonsignificant (*p* > 0.05) elevation in creatinine concentration in groups 4 and 5 compared to the standard group as presented in Table 5.

### 3.7. Effect of MESL on Mean Serum Superoxide Dismutase, Catalase, Glutathione Peroxidase Activities, and Malondialdehyde Concentrations of Treated Albino Wistar Rats

Groups three to six showed a nonsignificant (*p* > 0.05) difference in SOD activity compared to the treatment control. Groups four to six showed a significant (*p* < 0.05) increase in catalase activity compared to treatment group two. Groups three to six indicated a significant (*p* < 0.05) increase in glutathione peroxidase activity when compared to the treatment control. Groups three to six demonstrated a significant (*p* < 0.05) decrease in malondialdehyde levels compared to treatment control as shown in Table 6.

## 4. Discussion

Result of OGTT as reported in Table 1 indicated that the crude extract reduced the blood glucose of Wistar albino rats in significant manner. (*p* < 0.05) The extract showed substantial antidiabetic potency as corroborated in Table 2. There have been earlier reports of the hypoglycaemic potency of *S. longepedunculata* root bark and the use of the plant in managing diabetes mellitus in ethno-medicine. It is believed that different phytochemicals such as alkaloids, and flavonoids among others might account for this potency [17, 31]. In addition to the medicinal potentials of the root extract, the leaves are eaten as a vegetable [32, 33]. The antidiabetic and hypoglycaemic effects demonstrated by this work suggested that integrating moderate quantities of the leaves of this plant in the soup or sauce of persons with types 11 diabetes mellitus especially could sufficiently manage the disorder.

The results of liver function tests suggested propensity of MESL to elicit some hepatotoxicity at relatively high doses. These observations were partly in agreement with the findings of earlier researchers on some other extracts of the plant [32–34] Toxicity of *S. longepedunculata's* could arise from the fact that it contains methyl salicylate which is a known analgesic with serious toxicity at certain levels or dosages [35]. Relative efficient conjugation of bilirubin observed in this study suggested intact functioning of hepatocytes, toxic exposure notwithstanding.

The observed decrease in serum sodium ion might be a result of a possible symporter that sodium ion and glucose have. Sodium and glucose are known to have common co-transporter. Sodium-glucose co-transporter enables sodium ions and glucose molecules to get transported concomitantly across the membrane into the cell milieu. Potassium ion (K^+^) is a counter-ion to sodium ion (Na^+^). The nonsignificant deviation in potassium ion concentration observed suggested stability of the membranes of the nephrons. The overall stability of bicarbonate ion, potassium ion, and chloride ion concentrations suggested that there were relatively little or no perturbances to serum electrolytes thereby eliminating the possible aggravation of diabetes mellitus as seen in diabetic acidosis and alkalosis. Diabetes mellitus seemed to increase the serum concentrations of creatinine and urea. This might be a result of increased de-amination that amino acids face in diabetic conditions. Antidiabetic agents, therefore, are known to reverse these elevations. In an earlier study, increased serum urea and creatinine observed in untreated diabetic rats were reversed by antidiabetic principles like glibenclamide and *Cochlo spermum splanchnic* aqueous root extract [36]. This could account for the observed trend of the reversal of increased urea and creatinine levels of treated groups compared to the treatment control.

The observations of the in vivo antioxidants suggested that treatment had a protective effect on possibly the relevant pancreatic cells and different cell membranes though a redox imbalance was seen [32]. The varied antioxidant potencies seen could be accounted for by the strength of the different radicals that were scavenged. Superoxide ion is highly reactive, unlike hydrogen peroxide which is scavenged more easily by catalase and glutathione peroxidase [37].

## 5. Conclusion

The MESL caused meaningful reduction in blood glucose, some degree of hepatotoxicity and relatively nephron-safety, and moderate antioxidant rejuvenation potency. Therefore, caution should be exercised in order not to exceed the safe dose of MESL.

## Figures and Tables

**Table 1:** Effect of methanol extract of *Securidaca longepedunculata* leaves on the mean oral glucose tolerance test (OGTT).

Groups (mg/dL)	FBS	RBS 30 mins	RBS 60 mins	RBS 120 mins	RBS 120 FBS
Group 1	71.75 ± 4.57^a^	135.75 ± 14.17^a^	140.25 ± 14.67^a^	112 ± 8.20^a^	40.25 ± 8.19^a^
Group 2	98.25 ± 2.46^a,b^	144.5 ± 20.26^a^	131.75 ± 13.34^a^	121.5 ± 3.93^a^	23.25 ± 3.35^a,b^
Group 3	95.67 ± 3.38^a,b^	131.67 ± 10.53^a^	125.33 ± 7.88^a^	100.67 ± 6.33^a^	5 ± 5.29^b^

*Note:* Superscripts that have the same alphabets are nonsignificant while the ones with different alphabets are considered significant. Group 1 control normal saline 3 mL/kg BW unchallenged (no alloxan). Group 2 crude MESL 200 mg/kg BW prechallenged (using alloxan 100 mg/kg BW and allowed to normalize). Group 3 crude MESL 400 mg/kg BW prechallenged (using alloxan 100 mg/kg BW and allowed to normalize).

**Table 2:** Effect of methanol extract of *Securidaca longepedunculata* leaves on mean glucose concentration (mg/dL).

Groups	FBS	RBS day 0	RBS day 3	RBS day 6	RBS day 9
Group 1	78.00 ± 3.21	111.60 ± 3.16^a^	105.20 ± 5.82^a^	101.60 ± 3.57^a^	89.40 ± 4.52^a^
Group 2	75.00 ± 3.94	346.50 ± 51.27^a,b^	378.50 ± 39.18^a,b^	224 ± 46.85^b^	260.25 ± 20.46^b^
Group 3	89.50 ± 6.44	420.75 ± 59.67^a,b^	361.25 ± 41.79^a,b^	152.50 ± 31.10^a,b^	106.25 ± 19.25^a,b,c^
Group 4	89.00 ± 2.04	316.25 ± 36.70^a,b^	174.50 ± 53.93^a,b,c^	104.75 ± 9.77^a,b^	155 ± 27.89^a,b^
Group 5	86.25 ± 4.21	306.25 ± 83.26^a,b^	289 ± 94.11^a,b^	161.50 ± 23.18^a,b^	163.50 ± 75.57^a,b^

*Note:* Superscripts that have the same alphabets are nonsignificant while the ones with different alphabets are considered significant. Group 1: no induction + 3 mL/kg BW (normal control). Group II: induced with alloxan, but no treatment. Group III: induced with alloxan + 5 mg/kg BW of glibenclamide. Group IV induced with alloxan + 100 mg/kg BW of MESL. Group V: induced with alloxan + 200 mg/kg BW of MESL. Group VI: induced with alloxan + 400 mg/kg BW of MESL.

**Table 3:** Effect of methanol extract of *Securidaca longepedunculata* leaves on the mean serum bilirubin concentrations.

Groups	Mean Tbil Conc (mg/dL)	Mean Conj.bil Conc (mg/dL)
Group 1: no induction + 3 mL/kg BW (normal control)	2.100 ± 0.33^a^	0.629 ± 0.10^a^
Group II: induced with alloxan, but no treatment	0.860 ± 0.05^b^	0.665 ± 0.04^b^
Group III: induced with alloxan + 5 mg/kg BW of glibenclamide	2.030 ± 1.025^a,b^	0.965 ± 0.12^b,c^
Group IV induced with alloxan + 100 mg/kg BW of MESL	2.260 ± 0.76^a,b^	1.160 ± 0.24^c,d^
Group IV induced with alloxan + 200 mg/kg BW of MESL	2.420 ± 0.39^a,b^	1.370 ± 0.23^d^
Group VI: induced with alloxan + 400 mg/kg BW of MESL	2.360 ± 0.12^a,b^	0.883 ± 0.27^a,b,c^

*Note:* Superscripts that have the same alphabets are nonsignificant while the ones with different alphabets are considered significant.

**Table 4:** The effect of methanol extract of *Securidaca longepedunculata* leaves on the mean serum electrolyte concentrations.

Groups	Na^+^Conc (mMol/L)	K^+^Conc (mMol/L)	Cl^−^Conc (mMol/L)	HCO_3_^−^Conc (mMol/L)
Group 1:	124.40 ± 011.31^a^	2.640 ± 011.31^a^	119.800 ± 14.24^a^	19.800 ± 2.28^a^
Group II:	141.75 ± 1.71^b^	4.025 ± 0.76^b^	93.000 ± 5.83^a^	32.000 ± 2.94^b^
Group III:	137.25 ± 9.25^b^	3.750 ± 1.17^a,b^	109.900 ± 26.54^a^	22.450 ± 8.63^a,b^
Group IV	119.50 ± 10.66^a,b,c^	3.225 ± 0.79^a,b^	106.925 ± 13.51^a^	28.300 ± 9.15^a,b^
Group V:	147.33 ± 4.51^a,b,c,d^	3.300 ± 0.81^a,b^	113.133 ± 39.23^a^	24.867 ± 5.06^a,b^
Group VI:	136.50 ± 3.87^b^	3.200 ± 0.48^a,b^	92.250 ± 16.58^a^	22.500 ± 7.72^a,b^

*Note:* Superscripts that have the same alphabets are nonsignificant while the ones with different alphabets are considered significant. Group 1: no induction + 3 mL/kg BW (normal control). Group II: induced with alloxan, but no treatment. Group III: induced with alloxan + 5 mg/kg BW of glibenclamide. Group IV induced with alloxan + 100 mg/kg BW of MESL. Group V: induced with alloxan + 200 mg/kg BW of MESL. Group VI: induced with alloxan + 400 mg/kg BW of MESL.

**Table 5:** Effect of methanol extract of *Securidaca longepedunculata* leaves on mean serum urea and creatinine concentrations.

Groups	UreaConc (mg/dL)	CreatinineConc (mg/dL)
Group 1: no induction + 3 mL/kg BW (normal control)	32.60 ± 4.22^a^	1.080 ± 0.04^a^
Group II: induced with alloxan, but no treatment	135.250 ± 6.6^b^	5.460 ± 0.24^b^
Group III: induced with alloxan + 5 mg/kg BW of glibenclamide	63.000 ± 18.89^a,b^	1.760 ± 0.43^a,b^
Group IV induced with alloxan + 100 mg/kg BW of MESL	63.425 ± 13.48^a,b^	1.838 ± 0.45^a,b,c^
Group V: induced with alloxan + 200 mg/kg BW of MESL	59.733 ± 12.78^a,b^	1.813 ± 0.57^a,b,c^
Group V: induced with alloxan + 400 mg/kg BW of MESL	65.250 ± 34.22^a,b^	1.548 ± 0.71^a,b^

*Note:* Superscripts that have the same alphabets are nonsignificant while the ones with different alphabets are considered significant.

**Table 6:** Effect of methanol extract of *Securidaca longepedunculata* leaves on the mean serum SOD, catalase, GPx activities, and MDA levels.

Group	SOD (U/L)	CAT (U/L)	GPx (U/L)	MDA (mg/dL)
Group 1:	1.117 ± 0.02^a^	2.840 ± 0.39^a^	31.588 ± 3.69^a^	6.258 ± 0.19^a^
Group II:	1.134 ± 0.00^b^	1.378 ± 0.27^b^	12.425 ± 1.85^b^	8.768 ± 0.35^b^
Group III:	1.113 ± 0.02^a^	2.195 ± 0.85^b^	24.040 ± 8.12^a,b^	7.500 ± 1.47^a,b^
Group IV	1.099 ± 0.02^a^	3.356 ± 1.10^a,b,c^	28.920 ± 1.36^a,b^	6.875 ± 0.29^a,b^
Group V:	1.093 ± 0.01^a^	2.879 ± 0.19^a,b,c^	27.470 ± 2.77^a,b^	6.413 ± 0.17^a,b^
Group VI:	1.180 ± 0.03^a,b^	2.953 ± 0.90^a,b,c^	21.725 ± 5.68^a,b^	7.655 ± 1.08^a,b,c^

*Note:* Superscripts that have the same alphabets are nonsignificant while the ones with different alphabets are considered significant.

## Data Availability

All data generated from this study have been included in the manuscript.
